# A Polysorbate-Based Lipid Nanoparticle Vaccine Formulation Induces In Vivo Immune Response Against SARS-CoV-2

**DOI:** 10.3390/pharmaceutics17040441

**Published:** 2025-03-29

**Authors:** Aishwarya Saraswat, Alireza Nomani, Lin-Kin Yong, Jimmy Chun-Tien Kuo, Heather Brown, Muralikrishna Narayanareddygari, Avery Peace, Rizan Fazily, Timothy Blake, Christopher D. Petro, Bindhu Rayaprolu, Johanna Hansen, Amardeep Singh Bhalla, Mohammed Shameem

**Affiliations:** 1 Formulation Development Group, Regeneron Pharmaceuticals, Tarrytown, NY 10591, USA; aishwarya.saraswat@regeneron.com (A.S.);; 2 Vaccine Technology, Regeneron Pharmaceuticals, Tarrytown, NY 10591, USA; 3 Regeneron Genetic Medicines, Regeneron Pharmaceuticals, Tarrytown, NY 10591, USA; 4 Infectious Diseases, Regeneron Pharmaceuticals, Tarrytown, NY 10591, USA

**Keywords:** mRNA lipid nanoparticles, vaccines, Polysorbate-80, SARS-CoV-2, gene therapy, storage stability, immunogenicity

## Abstract

**Background:** Lipid nanoparticles (LNPs) have proven effective in delivering RNA-based modalities. Rapid approval of the COVID-19 vaccines highlights the promise of LNPs as a delivery platform for nucleic acid-based therapies and vaccines. Nevertheless, improved LNP designs are needed to advance next-generation vaccines and other gene therapies toward greater clinical success. Lipid components and LNP formulation excipients play a central role in biodistribution, immunogenicity, and stability. Therefore, it is important to understand, identify, and assess the appropriate lipid components for developing a safe and effective formulation. Herein, this study focused on developing a novel Polysorbate-80 (PS-80)-based LNP. We hypothesized that substituting conventional linear PEG-lipids with PS-80, a widely used, biocompatible injectable surfactant featuring a branched PEG-like structure, may change the LNPs biodistribution pattern and enhance long-term stability. By leveraging PS-80’s unique structural properties, this study aimed to develop an mRNA-LNP platform with improved extrahepatic delivery and robust freeze/thaw tolerance. **Methods**: We employed a stepwise optimization to establish both the lipid composition and formulation buffer to yield a stable, high-performing PS-80-based SARS-CoV-2 mRNA-LNP (SC2-PS80 LNP). We compared phosphate- versus tris-based buffers for long-term stability, examined multiple lipid ratios, and evaluated the impact of incorporating PS-80 (a branched PEG-lipid) on in vivo biodistribution. Various analytical assays were performed to assess particle size, encapsulation efficiency, mRNA purity, and in vitro potency of the developed formulation and a humanized mouse model was used to measure its immunogenicity over six months of storage at −80 °C. **Results:** Replacing the standard 1,2-dimyristoyl-rac-glycero-3-methoxy polyethylene glycol-2000 (PEG-DMG) lipid with PS-80 increased spleen-specific expression of the mRNA-LNPs after intramuscular injection. Formulating in a tris-sucrose-salt (TSS) buffer preserved the LNP’s physicochemical properties and in vitro potency over six months at −80 °C, whereas a conventional PBS-sucrose (PSS) buffer was less protective under frozen conditions. Notably, TSS-based SC2-PS80 LNPs elicited potent humoral immunity in mice, including high anti-spike IgG titers and robust pseudovirus neutralization, comparable to freshly prepared formulations. **Conclusions:** A PS-80-based mRNA-LNP platform formulated in TSS buffer confers improved extrahepatic delivery, long-term frozen stability, and strong immunogenicity against SARS-CoV-2 following six months. These findings offer a promising pathway for the design of next-generation mRNA vaccines and therapeutics with enhanced stability and clinical potential.

## 1. Introduction

Gene therapy has drawn increasing attention over the past decade for its potential to treat both inherited and acquired diseases, including cancer and infectious disorders. Among the many approaches to gene therapy, lipid nanoparticles (LNPs) have emerged as effective and comparatively safe non-viral delivery vehicles [1]. Their clinical utility is highlighted by the success of multiple mRNA-LNP vaccines against COVID-19—most notably, Pfizer-BioNTech’s Comirnaty^®^/BNT162b, and Moderna’s Spikevax^®^/mRNA 1273, both of which have received full FDA and EMA approval. Also, Moderna recently secured FDA approval for an mRNA-based RSV vaccine (mRESVIA^®^/mRNA 1345) [2]. Before this wave of mRNA-LNP success, the first RNA-encapsulating LNP therapy (Onpattro^®^/Patisiran) was approved by the FDA to treat polyneuropathy caused by hereditary ATTR amyloidosis, laying a strong clinical foundation for the LNP platform [3].

Despite these achievements, several challenges continue to impede the full translational impact of mRNA-LNPs. For instance, nucleic acids (including mRNA) are prone to enzymatic degradation, and their uptake in target tissues is hampered by their large molecular weight and negative charge [4,5]. Many LNPs preferentially accumulate in the liver, meaning only a fraction of each dose reaches extrahepatic targets such as the spleen or lymph nodes which are critical sites for vaccine delivery. Moreover, long-term storage stability remains an obstacle and the LNP drug product must be protected from physical and chemical degradation to preserve its activity. Hence, it is pivotal to develop a formulation with suitable excipients to enhance both the activity and stability of LNPs.

Lipid components and formulation excipients are the most essential aspects of the LNP formulation design. Ionizable lipids (e.g., DLin-MC3-DMA), which can comprise ≥50% of the total lipid, are crucial for mRNA encapsulation and endosomal escape. Helper lipids (e.g., phospholipids and cholesterol) form a stable bilayer that supports LNP structure. Meanwhile, PEG-containing lipids, though used at the lowest molar percentage, have a significant effect on the physicochemical properties, shelf stability, and in vivo kinetics of LNPs [6,7,8]. Common PEG-lipids, such as DSPE-PEG2K or DMG-PEG2K, feature linear PEG chains of ~2 kDa. Additionally, ~30% of an mRNA-LNP is the aqueous phase, so the excipient system (buffer, ionic strength, cryoprotectants, pH, etc.) can profoundly affect LNP formation, size, structure, and overall quality [9,10].

In this study, we investigated Polysorbate-80 (PS-80), a branched PEG surfactant, as an alternative PEG-lipid in LNPs formulated for SARS-CoV-2 vaccine development. PS-80 contains branched PEG-like chains linked to mainly an oleic acid-based tail [11]. It is widely recognized as a biocompatible injectable excipient and is routinely used in protein-based therapeutics at low concentrations (<0.5% *w*/*v*) to prevent aggregation [12,13,14].

One of the key objectives of our research was to employ PS-80 to replace widely used PEG-lipids and subsequently study the physicochemical properties, stability, and biodistribution of RNA-LNPs. We evaluated the in vivo biodistribution of PS-80-based LNP formulation containing Firefly Luciferase reporter mRNA (Fluc-PS80 LNP) in comparison to the standard DMG-PEG2K-based LNPs when formulated in PBS buffer. Our results showed preferential LNP accumulation in the spleen for Fluc-PS80 LNP when compared to DMG-PEG2k LNP following intramuscular injection. We then studied the effect of formulation excipients (including cryoprotectant, salt, lipid ratios, buffer composition as formulation factors) and developed a suitable Tris Sucrose Saline (TSS)-based formulation buffer to improve the physicochemical properties and potency of Fluc-PS80 LNP formulation.

Subsequently, we assessed the therapeutic potential of this PS-80-based formulation platform by encapsulating SARS-CoV-2 spike mRNA (SC2-PS80 LNPs) and demonstrated its improved long-term storage stability in comparison to the conventional PBS-sucrose (PSS buffer). Following six months of storage at −80 °C, the in vivo potency of SC2-PS80 LNPs was evaluated in a COVID-19 disease model. Our results demonstrated high immunogenicity of SC2-PS80 LNPs in genetically humanized VelocImmune^®^ mice [15]. Overall, we show that incorporating PS-80 as a branched PEG-lipid in an mRNA-LNP formulation resulted in enhanced spleen-targeted delivery as compared to the DMG-PEG2k LNPs and demonstrated acceptable storage stability in the TSS buffer. We hypothesize that PS-80’s branched PEG-like structure can influence particle biodistribution, potentially improving extrahepatic (e.g., spleen) uptake. Moreover, its surfactant properties are known to mitigate particle aggregation of biologics under stress conditions, suggesting that replacing standard PEG-lipids with PS-80 could enhance mRNA-LNP stability while retaining or even boosting vaccine potency. Our data show that this unique formulation elicited robust immune responses against SARS-CoV-2 in the humanized mouse model.

## 2. Materials and Methods

### 2.1. Materials

DLin-MC3-DMA (CAS# 1224606-06-7) was acquired from BioFine International Inc. (Vancouver, BC, CA). DSPC (CAS# 816-94-4), cholesterol (CAS# 57-88-5) and DMG-PEG2K (CAS# 880-15-1) were procured from Avanti Polar Lipids, Inc. (Alabaster, AL, USA). Polysorbate-80 (PS-80, CAS# 9005-65-6), ethyl alcohol (200 Proof) (CAS# E7023) was bought from Sigma-Aldrich, Inc. (St. Louis, MO, USA). CleanCap^®^ Firefly Luciferase (FLuc) mRNA (CAS# L-7602) was bought from TriLink BioTechnologies (San Diego, CA, USA). Quant-it^TM^ RiboGreen RNA Assay Kit (CAS# R11490), sodium acetate (CAS# J63669.AK), molecular biology grade water (CAS# J71786.XCR), PBS (CAS# 14190144), and other cell culture reagents were purchased from Thermo Fisher Scientific (Fair Lawn, NJ, USA). Other solvents, chemicals and reagents were obtained from VWR International (Radnor, PA, USA).

### 2.2. Cell Culture

HEK-293 cells (CRL-1573) were purchased from ATCC (Manassas, VA, USA) and cultured in Dulbecco’s Modified Eagle’s Medium (DMEM, CAS#11995073 supplemented with 10% heat-inactivated fetal bovine serum (FBS) and 1× penicillin/streptomycin. The cells were maintained in an atmosphere of 5% CO_2_ at 37 °C.

### 2.3. In Vitro Transcription of SARS-CoV-2 mRNA

Gene expressing SARS-CoV-2 6P [16,17] was cloned into a plasmid backbone, the coding sequence is flanked by 5′UTR and 3′UTR sequences. A T7 promoter is encoded upstream of the 5′UTR to initiate transcription using a T7 RNA polymerase. This promoter is compatible with CleanCAp AG reagent (TriLink BioTechnologies, San Diego, CA, USA) for co-transcriptional addition of the 5′ CAP during the in vitro transcription (IVT) reaction. The plasmid has a 104 nt segmented polyA tail that ends with a restriction site to allow for linearization of the plasmid at the end of the polyA tail. Plasmid was transformed into NEB stable *E. coli* cells and a colony was picked up and grown in LB media with 100 ug/mL ampicillin. Plasmid maxi prep was generated at our core facility and the sequence was verified using Sanger sequencing.

Further to this, mRNA was synthesized in an in vitro transcription reaction using T7 RNA polymerase (New England Biolabs, Ipswich, MA, USA) and linearized plasmid as a DNA template. The transcription reaction was set up at 37 °C for 2 h in the presence of 5 mM each of ATP, CTP, GTP (New England Biolabs, Ipswich, MA, USA) and N1-methyl Pseudouridine triphosphate (TriLink BioTechnologies, San Diego, CA, USA), transcription buffer (40 mM Tris-HCl, 10 mM DTT, 2 mM Spermidine, 0.002% Triton and 16.5 mM Magnesium Acetate) and the reaction was supplemented with 4 mM Clean Cap AG reagent. After transcription, the DNA template was digested with TURBO DNase (Invitrogen, Waltham, MA, USA) for 30 min at 37 °C and cleaned up using RNAClean XP magnetic beads (Beckman Coulter Life Sciences, Jersey City, NJ, USA), RNA was eluted in the RNA storage solution (AM7000, Invitrogen). The synthesized mRNA was quantified using the Qubit^TM^ RNA Extended Range (XR) Assay Kits (Invitrogen), and the integrity of the mRNA was evaluated on the 2100 Bioanalyzer instrument using the Agilent RNA 6000 Nano kit.

### 2.4. Formulation Development of Fluc-PS80 LNPs

#### 2.4.1. Cryoprotectant Concentration Screening in PSS Buffer

**Figure 1:**
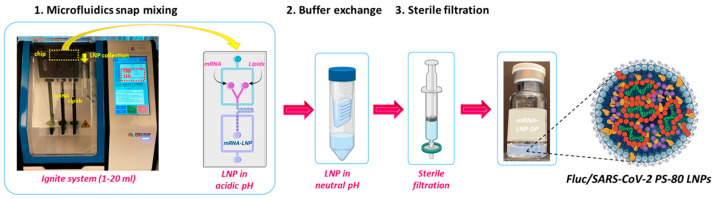
A schematic representation of the LNP manufacturing process using the NanoAssemblr^®^ Ignite^TM^ system. Constituent lipids and mRNA (acidic pH) are subjected to microfluidic mixing followed by buffer exchange using the formulation buffer for pH neutralization. The obtained neutral LNP solution is then sterile-filtered to produce the final mRNA-LNP DP. mRNA: Fluc or SARS-CoV-2; Lipids: DLin-MC3-DMA, DSPC, Cholesterol, PS-80; DP: drug product.

Fluc-PS80 LNPs were prepared via microfluidic mixing using the NanoAssemblr^®^ Ignite^TM^ system (Precision NanoSystems Inc., Vancouver, BC, Canada) (Figure 1). A freeze/thaw study was performed to screen varied concentrations of sucrose as the cryoprotectant to maintain the stability of LNPs. Fluc-PS80 LNPs were prepared with the lipid composition of DLin-MC3-DMA/DSPC/Cholesterol/PS-80 in the molar percent ratio of 52/8/37/3. The lipid components were dissolved in 200 Proof absolute ethanol at a total lipid concentration of 50 mM, while Fluc mRNA (200 µg/mL) was diluted in 10 mM sodium acetate buffer pH 4.5. Lipids and Fluc mRNA solutions were both mixed at the previously optimized flow rate ratio of 3:1 (aqueous/organic), total flow rate of 12 mL/min, and N/P ratio of 14. Formulated LNPs were diluted with 1× PBS, pH 7.4, containing various sucrose concentrations (0, 5, 10, 15, 20% *w*/*v*). Diluted Fluc-PS80 LNPs were subjected to ultrafiltration for buffer exchange using Amicon Ultra centrifugal filter units (100 kDa MWCO) (Millipore, Burlington, MA, USA). All the formulations were sterile-filtered, filled in 2R vials, and subjected to one freeze/thaw cycle (overnight at −80 °C). Detailed analytical characterization was performed for the obtained Fluc-PS80 LNPs as described in Section 2.5.

#### 2.4.2. Lipid Combination Screening in TSS Buffer

We performed the lipid combination screening for Fluc-PS80 LNPs in TSS buffer. This TSS buffer was developed in a preliminary study (see Supplementary Information, Figures S1 and S2 for more details). Different lipid combinations were screened by modifying the molar ratios of the four lipid components in the Fluc-PS80 LNP formulation. Briefly, LNPs containing different lipid combinations (shown in Table 1) were prepared using the NanoAssemblr^®^ Ignite^TM^ system (Precision NanoSystems Inc., Vancouver, BC, Canada). All six formulations (F1–F6) were prepared using similar mixing conditions described in Section 2.4.1. Following microfluidic mixing, LNPs were diluted with TSS buffer pH 7.4, and then subjected to buffer exchange using ultrafiltration followed by sterile filtration before filling into 2R vials. LNPs were tested in both frozen (one freeze/thaw cycle overnight at −80 °C) and liquid state (5 °C) to identify a stable formulation for further studies.

#### 2.4.3. Formulation Buffer Screening for Long-Term Storage Stability of SC2-PS80 LNPs

SC2-PS80 LNPs containing the optimized lipid composition of DLin-MC3-DMA/DSPC/Cholesterol/PS-80 in the molar percent ratio of 52/8/37/3 and sucrose content (identified from the cryoprotectant screening study) were prepared using the same manufacturing method as explained in Section 2.4.1. LNPs prepared in the TSS buffer were tested for long-term storage in comparison to the PSS buffer at different temperatures (−80 °C, 5 °C, 25 °C) over six months of storage. Following microfluidic mixing, LNPs were diluted with either of the two buffers: PSS (1× PBS, 10% sucrose, pH 7.4) or TSS (50 mM Tris, 45 mM NaCl, 10% sucrose, pH 7.4). Diluted LNPs were subjected to buffer exchange using ultrafiltration, sterile-filtered, and filled into 2R vials. The SC2-PS80 LNP samples were taken at various timepoints over 6 months for detailed characterization according to the analytical panel explained in Section 2.5.

### 2.5. Analytical Characterization of mRNA-Loaded LNPs

A panel for testing various quality attributes was established to identify the drug product (DP) quality of all the manufactured mRNA-LNP formulations using various analytical techniques/assays as mentioned in Table 2 and the following sub-sections.

#### 2.5.1. Visual Inspection and pH

Fluc-PS80 LNP formulations prepared for lipid combinations screening in TSS buffer (−80 °C, 5 °C, and 25 °C) were assessed for their appearance in terms of color, clarity, and visible particulates by visual observation against a black background with white light illumination. Additionally, to measure the pH, 100 µL of LNPs were aliquoted in 0.5 mL Eppendorf tubes and the small pH meter probe (Mettler Toledo) was immersed in the LNP solution to read the final pH following buffer exchange and sterile filtration. Visual inspection and pH measurement for LNP formulations subjected to long-term storage stability were determined at D0, D30, D90, and D180 at different storage conditions (−80 °C, 5 °C, 25 °C).

#### 2.5.2. Particle Size and Surface Charge

LNPs were analyzed for their particle size, polydispersity index by dynamic light scattering (DLS) and zeta potential using the electrophoretic mobility approach (Malvern Zetasizer Ultra). All the samples were measured in a disposable folded capillary cell (DTS1070) following 10 times dilution with sterile filtered 0.1× PBS, pH 7.4. Measurements were performed at 25 °C using a refractive index of 1.45 and an absorption of 0.001, with a viscosity of 0.8872 cP. Every sample was measured in triplicates, each with 5 runs and 5 s equilibration time, using a 173° backscatter detection angle.

#### 2.5.3. Particle Concentration

Fluc-PS80 LNP formulations prepared for lipid combinations screening in TSS buffer (−80 °C, 5 °C) were assessed for their particle concentration using a nanoparticle tracking analysis (NTA) instrument, ZetaView^®^ Z-NTA (Particle Metrix Inc., Mebane, NC, USA). Before running the LNPs, 100 nm polystyrene beads (AlphaNanoTech, Durham, NC, USA) were used for instrument QC, calibration, and auto-alignment. Then, LNPs were diluted between a range of 200,000×–500,000× in sterile-filtered 0.1× PBS pH 7.4 and injected into the cell for performing the measurements. Dilutions of each sample were identified to maintain 100–200 particles per frame to achieve accurate results. Particle count was performed for 11 positions/frames and three cycles, with a sensitivity of 85, shutter of 150, and laser wavelength of 488 nm for all the samples.

#### 2.5.4. Encapsulation Efficiency

A Quant-IT^TM^ RiboGreen RNA Assay Kit (Invitrogen^TM^) was used to measure the encapsulation efficiency and mRNA concentration of Fluc-PS80 LNP and SC2-PS80 LNP formulations. Initially, two standard curves were generated for Fluc and SARS-CoV-2 mRNAs (50–200 ng/mL), each in 10 mM Tris/1 mM EDTA (TE) buffer and in 2% *w*/*v* Triton x-100/TE (TR) buffer. The total mRNA in the LNPs was diluted 5× in TE buffer and added in a 1:1 volume ratio to TE buffer in each well to determine the free mRNA concentration. Next, to analyze the total mRNA concentration, the LNPs were diluted 25 times in TE buffer and added in a 1:1 volume ratio to TR buffer in each well. Following the addition of samples and standards to the plate, it was incubated at 37 °C for 10 min to allow LNP dissociation and subsequent release of the total encapsulated mRNA for quantification. Ribogreen reagent diluted in TE buffer (1:200 *v*/*v*) was then added to each standard and sample well in a 1:1 volume ratio and the plates were immediately measured for fluorescence (Ex485/Em528) using the Synergy Neo2 Hybrid Multimode Reader (Biotek, Shoreline, WA, USA). Free and total mRNA concentrations were calculated using the two standard curves generated in TE and TR buffers, respectively. The encapsulation efficiency for each LNP formulation was calculated using the following equation:

$$Encapsulation\;efficiency\;\left(\%\right)=\frac{Total\;mRNA-Free\;mRNA}{Total\;mRNA}\times 100$$

#### 2.5.5. mRNA Purity

Microchip capillary electrophoresis (MCE) was performed to determine the mRNA purity for all the tested LNP formulations. Fluc and SARS-CoV-2 mRNAs and their respective LNP samples were diluted with 2% Triton x-100 (*w*/*v* in nuclease-free water) to achieve a final concentration of 5 µg/mL. Next, 20 µL of the diluted samples were added to 80 µL of 1× Pico Sensitivity Sample Buffer into each well in a 96-well plate. Samples were denatured in a 70 °C heating block for 2 min, then snap-cooled on ice for 5 min. The RNA Pico ladder, Gel-Dye solution, and RNA Labchip were prepared by following the RNA Pico Sensitivity Assay Quick Guide (Perkin Elmer, Shelton, CT, USA) without any modifications. Briefly, 4 µL of RNA Pico ladder was added to 116 µL 1× Sample Buffer and transferred to the provided ladder tube. The Gel-Dye solution was prepared by transferring 90 μL of RNA Dye Concentrate to one RNA Pico Gel Matrix vial, followed by mixing and centrifugation in a microtube spin filter. The RNA Labchip was thoroughly washed with nuclease-free water, following which the Gel-Dye solution and RNA Pico marker were added to the designated wells. LabChip GXII Touch (Perkin Elmer, Shelton, CT, USA) was used to run the assay, which is based on the principle of mRNA electrophoresis that migrates across the sieving gel matrix to separate by size. The resultant mRNA signal was monitored by fluorescent detection. For each electropherogram obtained from running the Fluc or SARS-CoV-2 mRNA and their respective LNP samples, the main peak and the fragments were calculated as a percentage of the total peak area. The mRNA purity for both mRNA alone and mRNA encapsulated within LNPs is calculated as the percent peak area of the main peak.

#### 2.5.6. Particle Morphology

Cryogenic transmission electron microscopy (cryo-TEM) was used to determine the effect of lipid combinations screening on the morphology of Fluc-PS80 LNPs prepared in TSS buffer (−80 °C, 5 °C). For that, the C-Flat 2/1 holey carbon, mesh copper grids (Electron Microscopy Sciences, Hatfield, PA, USA) were cleaned with a Solarus 950 Plasma Cleaner (Gatan, Pleasanton, CA, USA) or a PELCO easiGlow Glow Discharge Cleaning System (Ted Pella, Inc, Redding, CA, USA) before sample application. A Vitrobot Mark IV (Thermo Fisher Scientific, Waltham, MA, USA) with the environmental chamber set to 4 °C and 95% or 100% humidity was used to prepare the grids. A 3 µL sample in the original TSS buffer composition was applied to a grid for up to 30 s before blotting and plunge freezing in liquid ethane. Grids were stored under liquid nitrogen, clipped into cartridges, placed into a cassette, and transferred to the autoloader of the microscope while maintaining cryogenic temperatures (below −170 °C). Images were collected with a Glacios Cryo-Transmission Electron Microscope (Thermo Fisher Scientific, Waltham, MA, USA) equipped with a Falcon 3 or Falcon 4 camera (Thermo Fisher Scientific, Waltham, MA, USA) and operated at 200 kV. Leginon software (version 3.7) was used for semi-automated data collection. Areas of interest were targeted at lower magnifications (940×–8500×) before the acquisition of movies at nominal magnifications of 28,000× (~0.5 nm/pixel), 73,000× (~0.2 nm/pixel), and 150,000× (~0.1 nm/pixel). The 150,000× images were acquired with defocus between −2 µm to −4.5 µm and with an electron dose between 12–25 e−/Å2. Finally, the acquired movie frames were aligned with MotionCor2 (Version 1.6.4) and converted to a jpeg format.

### 2.6. In Vitro Transfection Efficiency of Fluc-PS80 LNP Formulations

The in vitro luciferase expression assay was performed in HEK-293 cells. Briefly, cells were seeded at a density of 10,000 cells per well in 96-well plates 24 h prior to the treatments. Following that, cells were treated with Fluc-PS80 LNPs containing different lipid combinations at 10 ng mRNA per well. Lipofectamine^TM^ MessengerMAX^TM^ Transfection Reagent (Invitrogen, Cat# LMRNA015) was used as a positive control, by following the manufacturer’s instructions. After 24 h of treatment, ONE-Glo^TM^ Luciferase Assay (Promega, Madison, WI, USA) was performed as per the manufacturer’s protocol. For that, an equal volume of the assay reagent was added to each treatment and control wells and incubated for 3 min at room temperature. Following this step, 150 µL of the supernatant was transferred to a white opaque 96-well microplate and the luminescence was measured on a Tecan Infinite F500 Multimode Microplate Reader (Thermo Fisher Scientific, Waltham, MA, USA).

### 2.7. In Vivo Transfection of Fluc-PS80 LNP Versus DMG-PEG2K LNP Formulations

All animal studies were conducted according to Regeneron’s and Institutional Animal Care and Use Committee (IACUC) for murine research (Protocol 303.4). The animal handling was performed in facilities accredited by the Association for Assessment and Accreditation of Laboratory Animal Care International in accordance with protocols approved by IACUC and the principles outlined in the Guide for the Care and Use of Laboratory Animals by the Institute for Laboratory Animal Resources. The 4–8-weeks-old, wild-type C57BL/6NTac female mice (Taconic Biosciences, Inc., Rensselaer, NY, USA) were IV (tail vein) or IM (hind limb) injected with 12 µg mRNA per mouse from Fluc mRNA formulated with PS-80 LNP or with conventional LNPs containing D-Lin-MC3-DMA and DMG-PEG lipids (Table 3). We used PBS solution to dilute the LNP samples and the maximum injection volume of samples was less than 100 µL per injection. Each treatment group contained four mice with an additional untreated mouse kept in the same cage as the control. The mice’s whole-body images were collected 24 h post-LNP injection using IVIS. Before IVIS imaging, 100 µL of D-Luciferin substrate was injected per mouse. The mouse was maintained under 2% isoflurane anesthesia for 10 min. After whole-body imaging, the mice were sacrificed by exposing them to CO_2_ gas for 10 min, and then their internal organs, including the liver, spleen, and lymph nodes, were dissected and subsequently imaged under the IVIS instrument.

### 2.8. In Vitro Transfection Efficiency of SC2-PS80 LNP Formulations

#### 2.8.1. Transfection of HEK-293 Cells with SC2-PS80 LNPs

The day before transfection, HEK-293 cells were seeded at 10,000 cells per well of a flat bottom 96-well plate and left to adhere overnight. Next, SC2-PS80 LNPs formulated in different buffers and stored at various temperatures were diluted with complete media to make 1 µg/mL mRNA, then added to HEK-293 cells in the 96-well plate. For the positive control, cells were transfected with mRNA formulated with Lipofectamine^TM^ MessengerMAX^TM^ (Invitrogen, Cat# LMRNA015), prepared according to the manufacturer’s instructions. The resulting LNPs and Lipofectamine lipoplexes were added to the HEK-293 cells at 1/10th the media volume of the cells, at 1 µg/mL final mRNA concentration. After that, cells were incubated for 18–24 h at 37 °C, 5% CO_2_ before being harvested to measure the expression of SARS-CoV-2 6P spike protein using flow cytometry.

#### 2.8.2. Immunostaining of Transfected Cells and Analysis of SARS-CoV-2 6P Spike Protein Expression via Flow Cytometry

The transfected HEK-293 cells were washed by Mg/Ca-free DPBS (Gibco Cat# 14190144) then trypsinized, re-washed and transferred to a round bottom 96-well plate. Next, to measure the viability, 50 µL DPBS containing Live/Dead Fixable Violet (Invitrogen, Cat# L34964) viability dye was added to each well and incubated for 15 min at 37 °C in the dark. After that, the cells were washed with DPBS twice, and the primary antibody specific for SARS-CoV-2 6P spike protein was added to the cells at 4 µg/mL in 50 µL cell staining buffer (DPBS containing 1% FBS and 0.5 mM EDTA) and incubated for 20–30 min at room temperature in the dark. Cells were washed twice with DPBS, and an anti-human FcR secondary antibody conjugated to Alexa Fluor 647 (AF647) (Jackson Immunoresearch, Cat# 109-605-190) was added to the cells at 2 µg/mL in 50 µL cell staining buffer and incubated for 20–30 min at room temperature in the dark. Finally, the cells were washed twice with DPBS before resuspending in 80 µL of cell staining buffer for flow cytometry analysis on the Agilent Novocyte Penteon flow cytometer (Agilent Technologies, Santa Clara, CA). For flow cytometry, a minimum of 20,000 events were recorded and exported to FCS 3.0 files to be analyzed by the FlowJo software (version 9.9.6, BD, Ashland, OR) to quantify the expression of SARS-CoV-2 6P spike protein via AF647 gating. The Live/Dead Violet-negative and AF647-positive gating were used to quantify using FlowJo software. The level of spike protein expression was quantified by either the median fluorescence intensity (MFI) of AF647 in live cells, or the percentage of live cells that expressed AF647 relative to non-transfected cells.

### 2.9. Mouse Immunization and In Vivo Immunogenicity of the Optimized SC2-PS80 LNP Formulation

VelocImmune^®^ mice (8–20 weeks of age) were immunized IM into the hind limb with 5 µg/mouse at 50 μL total volume diluted in PBS of SARS-CoV-2 mRNA encoding Wuhan-Hu-1 SARS-CoV-2 spike sequence (MN908947.3 with K986P + V987P stabilizing mutations) and SC2-PS80 LNP formulation. The mRNA/LNPs were either made fresh (n = 5/group) or stored at −80 °C or 5 °C (n = 3/group) for 6 months prior to vaccination. Sera was collected 26 days post single immunization and stored at 4 °C. Luminex spike assay was performed following previously published methods for polyclonal SARS-CoV-2 specific IgG binding on an INTELLIFLEX^®^ Luminex^TM^ instrument [18]. Sera samples were diluted to 1:6250 and reported as median fluorescence index (MFI) via an anti-mouse IgG-PE secondary antibody. Sera binding was to full-length Wuhan-Hu-1 SARS-CoV-2 spike protein.

Pseudovirus neutralization assays were performed on samples following previously published methods using non-replicative vesicular stomatitis virus (VSV) with firefly luciferase (Fluc) encoded and SARS-CoV-2 spike (aa 14-1255, D614G substitution, Wuhan-Hu-1; MN908947.3) replacing the native VSV glycoprotein (G) sequence [18]. Luciferase output was measured on a SpectraMax i3 plate reader with a MiniMax imaging cytometer. Sera samples are reported as pseudotyped virus neutralization titer 50 (pVNT50).

### 2.10. Statistical Analysis

Statistical analyses were performed for all the experiments using GraphPad Prism software (version: 10.1.0). All data were presented as mean ± SD for the in vitro assays, while that as mean ± SEM for any in vivo experiments. A *p* value of less than 0.05 was considered statistically significant.

## 3. Results

### 3.1. Fluc-PS80 LNPs Preferentially Accumulate in the Spleen Rather than the Liver

**Figure 2:**
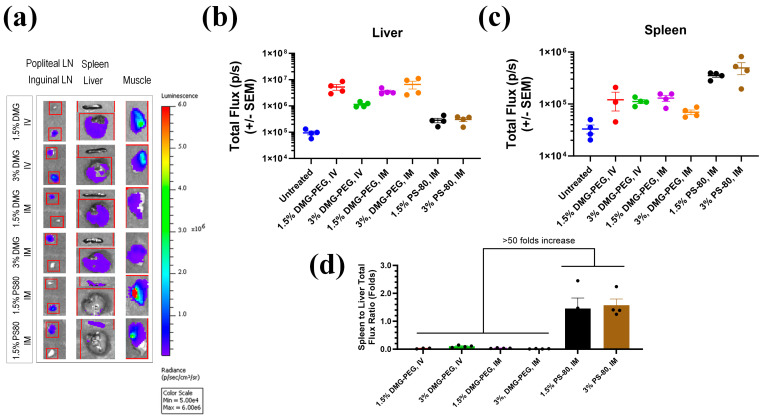
Organ transfection distribution of Fluc-PS80 LNPs compared to the DMG-PEG2K LNPs post-IV or IM administration in mice. (**a**) Luciferase expression of the dissected organs under IVIS; (**b**,**c**) quantified total flux for liver and spleen; and (**d**) Luciferase expression (total flux) fold ratios in the spleen compared to the liver. Four mice were injected per group and the data (**d**) were statistically compared using ANOVA with Bonferroni post hoc.

To gauge the effect of PS-80 on extrahepatic delivery, we formulated Fluc-PS80 LNPs at two PS-80 molar percentages (1.5% or 3%) in PBS buffer and compared their biodistribution with a conventional DMG-PEG2K LNP in mice (Table 3). All LNPs maintained a particle size below 100 nm and encapsulation efficiencies above 90%. We included IV administration of DMG-PEG LNPs as a baseline control to highlight the well-known liver accumulation typically seen with conventional PEG-lipid LNPs via IV injection. We showed that with the change of the administration route from IV to IM, the DMG-PEG2K LNPs’ liver accumulation pattern did not change. Following IM injection of ~12 µg mRNA per mouse, ex vivo IVIS imaging (Figure 2) revealed that independent of the PS-80 percentage, Fluc-PS80 LNPs yielded, on average, a 3-to-5-fold higher luciferase signal in the spleen relative to DMG-PEG2K formulations (Figure 2c). Moreover, the spleen-to-liver expression ratio exceeded 50-fold in the PS-80 groups (Figure 2d), suggesting substantially enhanced extrahepatic uptake. Based on these promising results, we next refined the formulation buffer, cryoprotectant concentration, and lipid composition, to then evaluate the long-term stability of the optimized PS-80 LNP platform for the SARS-CoV-2 spike mRNA vaccine.

### 3.2. Optimization of Formulation Composition for Fluc-PS80 LNPs

Before optimizing other formulation variables (e.g., cryoprotectant levels, buffer types, and lipid compositions), we first tested how salt concentration affects the freeze/thaw stability of Fluc-PS80 LNPs. We found that 45 mM NaCl was necessary to maintain consistent particle attributes and potency, particularly in tris-based buffer (Figure S3). Consequently, all subsequent development of cryoprotectant and lipid composition was performed in the presence of ≥45 mM NaCl.

#### 3.2.1. Buffer and Cryoprotectant Screening

**Figure 3:**
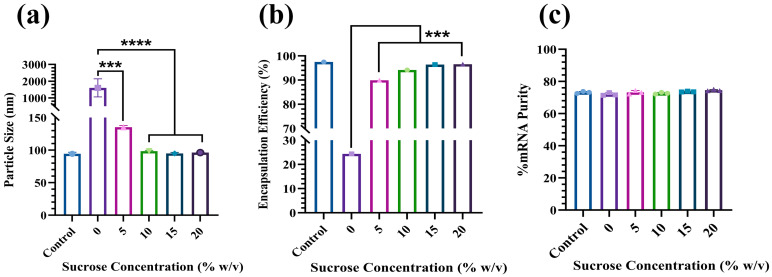
Screening of sucrose concentration to prepare stable Fluc-PS80 LNP formulation following one freeze/thaw cycle. LNP formulations containing ≥10% sucrose exhibited consistently smaller particle sizes of <100 nm, ≥90% encapsulation efficiency, and >70% mRNA purity. The control samples were the same LNPs stored at 5 °C overnight without the exposure to freeze/thaw cycle. The bars show the mean ± standard deviation of three independent measurements. Statistical comparison between samples was analyzed by performing one-way ANOVA with Bonferroni post hoc: *** *p* value < 0.001, **** *p* value < 0.0001.

Next, we screened sucrose levels ranging from 0% to 20% (*w*/*v*) in PBS (PSS) to identify the optimal cryoprotectant content for Fluc-PS80 LNPs under freeze/thaw stress. As shown in Figure 3, formulations containing ≥10% sucrose maintained a particle size ≤100 nm, encapsulation efficiency ≥90%, and mRNA purity higher than 70% following one freeze/thaw cycle (stored at −80 °C overnight and thawed at room temperature). By contrast, samples with <10% sucrose tended to aggregate or lose encapsulation upon the freeze/thaw cycle.

In a separate set of experiments, we observed that a tris-sucrose-salt (TSS) buffer system (50 mM tris, 45 mM NaCl, 10% *w*/*v* sucrose) provided even better long-term stability for Fluc-PS80 LNPs than PSS (see Supplementary Information, Figures S1 and S2). As a result, TSS was selected for further in vitro and in vivo evaluations.

#### 3.2.2. Lipid Combinations Screening

After establishing 10% sucrose in a tris-salt buffer (TSS) as optimal for freeze/thaw stability, we screened various PS-80 and helper-lipid ratios (Table 1) to determine their impact on Fluc-PS80 LNP quality and performance. Each formulation (F1–F6) was tested for physicochemical attributes (particle size, zeta potential, encapsulation efficiency, and particle concentration), then further examined via cryo-TEM and in vitro transfection.

##### Particle Size, Particle Concentration, and Encapsulation Efficiency

**Figure 4:**
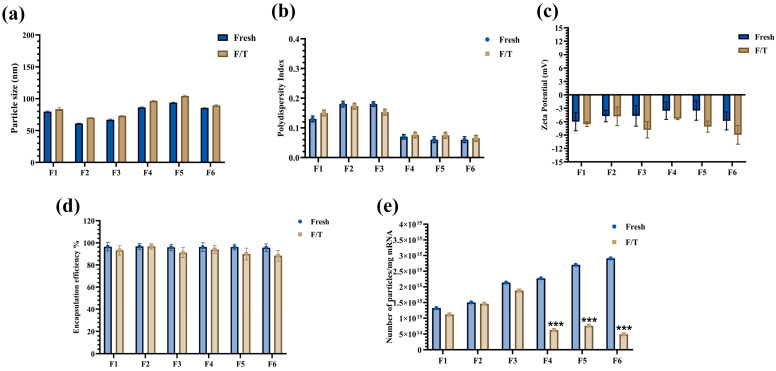
Physicochemical characterization of Fluc PS-80 LNP formulations containing various lipid combinations prepared in TSS buffer, including (**a**) Particle size, (**b**) Polydispersity index, (**c**) Zeta Potential, and (**d**) Encapsulation efficiency. F4–F6 formulations containing 1.5 mol% PS-80 demonstrated lower polydispersity index while the other analyses yielded similar results for all the formulations. (**e**) Particle concentration normalized to the mRNA content sharply reduced for F4-F6 formulations following freeze/thaw. The represented data are mean ± standard deviation of three independent measurements. Statistical comparison between samples was performed using one-way ANOVA with Bonferroni post hoc: *** *p* value < 0.001.

The particle size of all formulations was maintained below 100 nm pre- and post-freeze/thaw, albeit with different size distributions. Specifically, the polydispersity index (PDI) was lower (≤0.1) in F4–F6 (1.5% PS-80) compared to F1–F3 (3% PS-80), whose PDI was ≤0.2 (Figure 4a,b). Zeta potential remained near neutral (−3 to −10 mV), and encapsulation efficiencies exceeded 85% across all formulations, with no major changes from freeze/thaw (Figure 4c,d).

Despite these similarities, particle concentration differed markedly. Formulations F4–F6 lost 3- to 6-fold more particles upon one freeze/thaw than F1–F3 formulations did (Figure 4e). This suggests that lower PS-80 concentrations may yield narrower size distributions but could also compromise freeze/thaw robustness.

##### Particle Morphology by cryo-TEM

**Figure 5:**
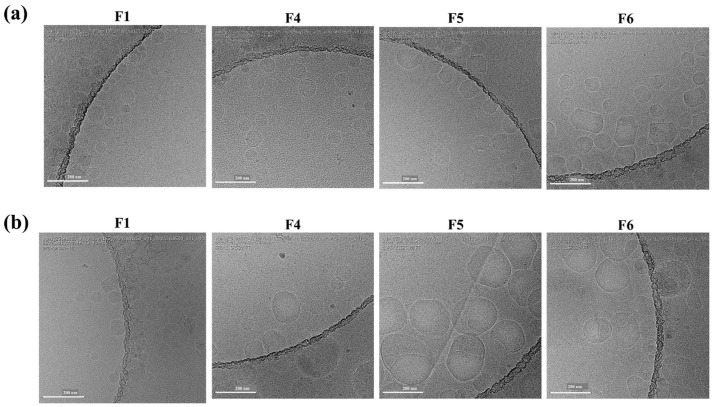
Representative cryo-TEM images of four Fluc-PS80 LNP formulations prepared in TSS buffer. (**a**) Fresh formulations, and (**b**) following one freeze/thaw cycle at −80 °C. For fresh formulations, F4–F6 formulations containing 1.5 mol% PS-80 showed a higher number of particles as compared to the F1 formulation containing 3 mol% PS-80. Increasing DSPC and simultaneously decreasing Dlin-MC3-DMA content resulted in changed particle morphology from circular to elongated LNPs with polyhedral shapes. For formulations subjected to freeze/thaw, F5 and F6 formulations showed a significant increase in the size of the particles as compared to the F1 formulation. The scale bar shows 200 nm for all presented images.

To visualize how lipid composition and freeze/thaw affected particle morphology, we examined F1 and F4–F6 by cryo-TEM (Figure 5). Both fresh F1 (3% PS-80) and F4 (1.5% PS-80) were generally spherical with minor internal blebs, suggesting PS-80 concentration alone did not drastically alter particle shape. However, increasing DSPC and reducing DLin-MC3-DMA (F5–F6) led to more elongated, polyhedral morphologies. After a single freeze/thaw cycle, the number of particles decreased for all samples, and F4–F6 displayed larger blebs with uneven surfaces—consistent with the observed decrease in particle concentration (Figure 4e). Considering the apparent particle sizes, it should be noted that Figure 4a shows the average hydrodynamic diameter in suspension (DLS), whereas cryo-TEM (Figure 5) images individual frozen particles, which can appear larger or more irregular than the DLS-measured mean size because the two techniques operate under different principles and conditions.

##### mRNA Purity and In Vitro Transfection

**Figure 6:**
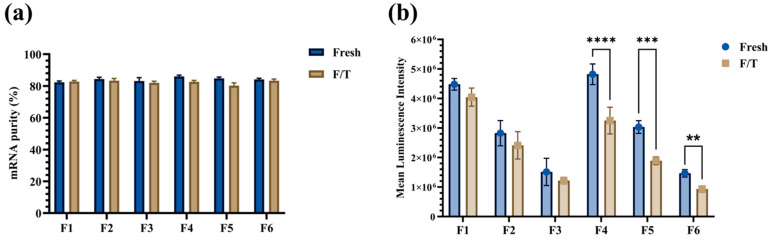
mRNA purity and in vitro transfection efficiency analyses of Fluc-PS80 LNP formulations containing various lipid combinations prepared in TSS buffer. (**a**) No substantial difference was observed between formulations in terms of mRNA purity, and (**b**) in vitro transfection efficiency was reduced with increased DSPC and decreased Dlin-MC3-DMA content. While F4–F6 formulations containing 1.5 mol% PS-80 showed higher transfection efficiency as compared to F1–F3 formulations, it significantly reduced following freeze/thaw. The formulations following freeze/thaw were significantly different in the mean luminescence intensity. The represented data are mean ± standard deviation of three independent measurements. Statistical comparison between samples was analyzed using Two-Way ANOVA with Bonferroni post hoc. ** *p* value <0.05, *** *p* value < 0.001, **** *p* value < 0.0001.

All LNPs preserved ≥80% mRNA purity, independent of formulation or freeze/thaw (Figure 6a). In contrast, in vitro transfection efficiency declined with rising DSPC and reduced DLin-MC3-DMA. Mean luminescence intensity dropped, for instance, from 4.48 × 10^6^ a.u. (F1) to 1.51 × 10^6^ a.u. (F3) in the 3% PS-80 series (Figure 6b). Additionally, LNPs with 1.5% PS-80 (F4-F6) suffered a further 1.5-fold reduction post-freeze/thaw, whereas 3% PS-80 formulations (F1–F3) remained unaffected. Overall, F1 offered the best balance of freeze/thaw stability and high transfection and was thus chosen to encapsulate SARS-CoV-2 mRNA for subsequent vaccine-related studies.

### 3.3. Storage Stability of SC2-PS80 LNPs In Tris vs. Phosphate-Based Buffers

**Figure 7:**
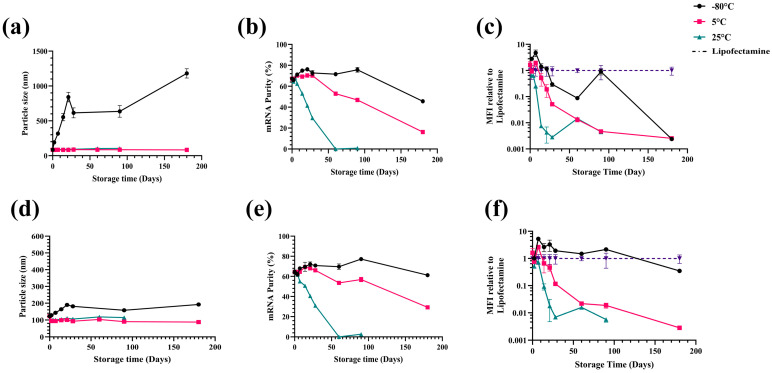
Storage stability of SC2-PS80 LNPs formulated in PSS (**a**–**c**) and TSS (**d**–**f**) buffers following six months when stored at different temperatures. SC2-PS80 LNPs formulated in PSS buffer and stored at −80 °C showed increased particle size and reduced mRNA purity as well as in vitro transfection efficiency. Most formulations depicted lower stability in terms of mRNA purity and in vitro transfection efficiency over time. SC2-PS80 LNPs formulated in TSS buffer and stored at −80 °C showed higher stability as compared to those prepared in PSS buffer with improved particle size, mRNA purity, and in vitro transfection efficiency over six months. The represented data are mean ± standard deviation of at least three independent measurements. MFI: Mean Fluorescence Intensity.

Having identified F1 as the lead composition, we next encapsulated SARS-CoV-2 mRNA and compared the long-term stability of SC2-PS80 LNPs in PBS-sucrose (PSS) versus tris-sucrose-salt (TSS) buffers at −80 °C, 5 °C, or 25 °C for up to six months (Figure 7).

#### 3.3.1. PSS-Formulated LNPs

SC2-PS80 LNPs in PSS showed significant instability when stored in a frozen state. Their particle size increased from ~85 nm to ~1180 nm at −80 °C, accompanied by an increase in polydispersity index (PDI) from 0.10 to 0.55 (Figure 7a and Figure S6). While samples stored at 5 °C or 25 °C retained smaller sizes (85–100 nm), they demonstrated a notable reduction in mRNA purity (16.2% and 79% by six and three months, respectively; Figure 7b) and showed a significant loss in their in vitro transfection efficacy over time (Figure 7c). Overall, PSS-buffered LNPs did not maintain adequate particle properties at the tested temperatures.

#### 3.3.2. TSS-Formulated LNPs

In contrast, TSS-based LNPs demonstrated substantially greater stability under frozen conditions. After six months at −80 °C, their size increased only to ~192 nm, with a PDI of ~0.13 (Figure 7d and Figure S6c). Zeta potential also remained stable across all temperatures (Figure S6). Although mRNA purity at 5 °C or 25 °C gradually dropped (to 29.2% and 2.6%, respectively), the −80 °C samples retained ~61.2% (Figure 7e). Importantly, TSS-based LNPs stored at −80 °C preserved high in vitro transfection, comparable to that of Lipofectamine (used as positive control) (Figure 7f).

To contextualize our PS-80 based formulation, we performed a parallel stability assessment of conventional DMG-PEG2K LNPs in PSS versus TSS buffers (see Supplementary Figures S1 and S2). Although DMG-PEG2K LNPs also benefited from TSS in maintaining smaller particle sizes and higher in vitro potency over six months, their particle size was less affected by the buffer type under frozen storage compared to the PS-80-LNPs. Hence, while both formulations gain stability from TSS, PS-80-LNPs offer an added advantage of enhanced extrahepatic (spleen) localization (Figure 2), making them an attractive platform for mRNA vaccine applications.

Taken together, these data confirm that tris-sucrose-salt (TSS)-buffered PS-80 LNPs provide robust particle size control, mRNA integrity, and in vitro activity over extended storage at −80 °C, whereas freezing in phosphate-salt-sucrose (PSS) buffer leads to aggregation and reduced potency.

### 3.4. In Vivo Immunogenicity of the Optimized SC2-PS80 LNP Formulation

**Figure 8:**
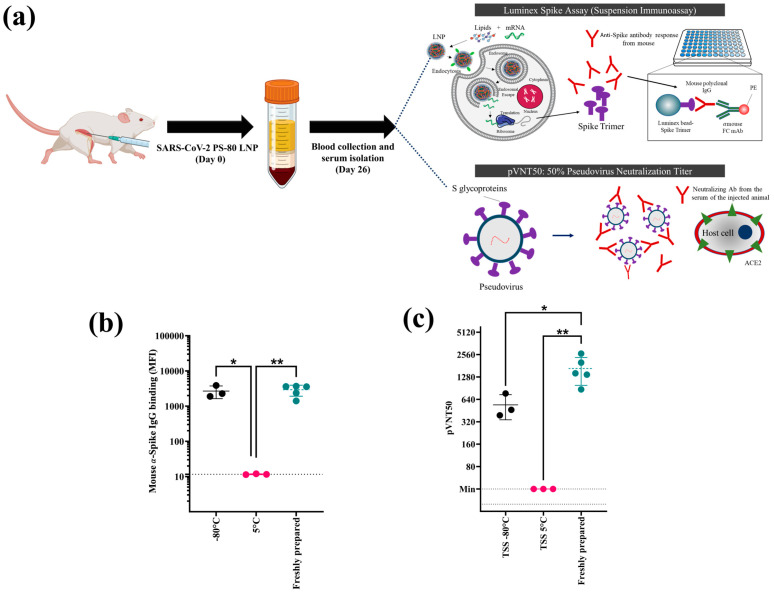
In vivo immunogenicity of the optimized SC2-PS80 LNPs in mice following six months of storage. (**a**) Schematics representing mice inoculation, sera collection and immunogenicity analyses following intramuscular administration of SC2-PS80 LNPs. (**b**) Anti-spike IgG antibodies were measured via a bead-based Luminex assay for spike in serum from mice immunized with the LNPs stored at −80 °C compared to the freshly prepared formulation at 1:6250 dilution of sera. (**c**) pVNT50 (50% pseudovirus neutralization titer) assay denoting the pVNT50 values of the formulation stored at −80 °C and 5 °C for 6 months in comparison to freshly prepared SC2-PS80 LNPs. The data on graphs (**b**) and (**c**) are mean ± standard deviations of at least three independent measurements. Statistical comparison between samples was analyzed using One-Way ANOVA with Bonferroni post hoc. * *p* value <0.1, ** *p* value < 0.05. The schematic was partially created with BioRender.com.

Following verification of the SC2-PS80 LNP’s in vitro potency and stability, we assessed its immunogenicity in either a frozen state (−80 °C) or a liquid state (5 °C) after six months of storage in TSS buffer. At 26 days post-single immunization, serum samples were collected and evaluated for SARS-CoV-2-specific IgG binding and neutralization (Figure 8a). Results were compared to freshly prepared LNPs to detect any changes in vivo efficacy over storage time.

Mice that received LNPs stored at −80 °C for six months produced anti-spike IgG titers on par with those given freshly prepared formulations (Figure 8b). By contrast, the group stored at 5 °C showed minimal IgG binding, consistent with the drop in transfection efficiency seen in vitro for 5 °C samples. Similarly, pseudovirus neutralization titers (pVNT50) were robust in both freshly prepared and −80 °C–stored LNPs (average 540 and 1664 a.u., respectively), while 5 °C-stored LNPs induced negligible neutralization (Figure 8c). These findings confirm that formulating in TSS buffer and storing at −80 °C preserves the vaccine’s ability to stimulate a strong SARS-CoV-2-specific humoral response.

## 4. Discussion

mRNA-LNPs have been extensively utilized as non-viral mRNA delivery systems both in preclinical and clinical studies given their safe and biodegradable nature, ease of manufacturing and scalability, low immunogenicity, as well as acceptable potency in delivering RNA therapeutics. LNPs have the capability to induce humoral and cellular immunity, which makes them an ideal carrier to fight against viruses that are easy to mutate. The recent approvals of Comirnaty^®^, Spikevax^®^, and mRESVIA^®^ are prime examples of how LNPs can be harnessed for vaccine development. Nonetheless, improving extrahepatic targeting and long-term stability remains a key challenge, particularly for formulations intended as vaccines rather than therapies targeted to the liver.

In this study, we explored Polysorbate-80 (PS-80) as a branched PEG-lipid, and fine-tuned the formulation excipients to address both biodistribution and storage limitations. PS-80 has been used as a stabilizer in some FDA approved vaccine formulations such as those for H5N1 influenza (Adjuvant System 03/AS03), HPV (GARDASIL 9), DTaP (Infanrix), DTaP-IPV (Kinrix and Quadracel), DTaP-HepB-IPV (Pediarix), DTaP-IPV/Hib (Pentacel), Hep B (Heplisav-B), Meningococcal B (Trumenba), PCV13 (Prevnar 13), Rotavirus (RotaTeq), and Tdap (Boostrix) [19,20,21,22,23,24,25]. The role of the PEG-lipid in stabilizing lipid nanocarriers like liposomes and LNPs is also demonstrated in the literature [8,26]. The role of the PEG-lipid in stabilizing lipid nanocarriers like liposomes and LNPs is clearly demonstrated in the literature [8,26]. However, the structural conformation and the shedding nature of the PEG and their effect on the LNP biodistribution are among the less evaluated characteristics of the LNPs. Our initial comparisons of Fluc-PS80 LNPs against conventional DMG-PEG2K LNPs revealed a pronounced shift in organ distribution, with PS-80-containing formulations displaying a three- to five-fold higher accumulation in the spleen and reduced liver uptake (Figure 2). DMG-PEG2K was chosen as a standard of comparison, given that it is one of the commonly used PEG-lipids for LNP formulations. Also, DMG-PEG2K is part of the marketed drug products like Spikevax^®^, Onpattro^®^, and mRESVIA^®^. Nevertheless, these findings suggest that modifying the PEG-lipid structure can meaningfully alter LNP tropism, thereby improving vaccine relevant delivery to immune organs such as the spleen and lymph nodes. Notably, imaged capillary electrophoresis (iCE) measurements (see the Supplementary File, Section 2.6 and Figure S7) revealed that DMG-PEG LNPs have a slightly higher isoelectric point (pI) than PS80-based LNPs (7.96 vs. 7.63), indicating that the net charge of LNPs might have contributed to the observed LNP’s biodistribution change. However, we speculate that alongside the lower net charge, PS-80’s branched PEG-like headgroup can additionally alter how serum proteins adsorb to the LNP surface. This modified protein corona may favor spleen localization over hepatic clearance. In addition, PS-80 LNPs might have a reduced rate or extent of opsonization due mainly to their closer to the neutral charge relative to PEG-DMG LNPs, thereby promoting extrahepatic uptake. This hypothesis aligns with other reports indicating that subtle differences in PEG-lipid structure can significantly impact biodistribution [27]. Future mechanistic studies, including in-depth protein corona analysis, may clarify how PS-80 promotes extrahepatic uptake and improves immune-targeted delivery.

Beyond biodistribution, formulation stability is also pivotal for the long-term storage of LNPs. While cryoprotectants like sucrose are already known to preserve LNP quality during freezing [28], our systematic screening identified 10% (*w*/*v*) sucrose and 45 mM salt as a required combination to maintain particle size (<100 nm), encapsulation efficiency (>90%), and mRNA integrity (>70%) of PS-80-based formulations after a single freeze/thaw. Tailoring lipid composition—i.e., both lipid type and concentration—is another critical aspect when optimizing mRNA-LNP formulations for stability and potency [29]. Previously, PS-80 was shown to stabilize lipoplex formulations at 3 mol% and enhance the solubilization of other lipid components [30]. Building on that, we screened various molar ratios of DLin-MC3-DMA (48–52%), DSPC (8–12%), cholesterol (37–38.5%), and PS-80 (1.5–3%) in Fluc-PS80 LNPs to explore how each lipid affects particle quality and in vitro activity. The concentration of each of these lipid components was maintained within the acceptable range so that their function in encapsulating or delivering the mRNA was not impacted. Our data indicated that 3 mol% PS-80 (F1–F3) yielded smaller mean diameters (60–80 nm) with PDI ≤ 0.2, whereas dropping to 1.5 mol% (F4–F6) increased particle size slightly (80–100 nm) yet narrowed the distribution (PDI ≤ 0.1). All formulations maintained neutral zeta potential, high encapsulation efficiency, and mRNA purity before and after freeze/thaw. However, transfection efficiency declined when DLin-MC3-DMA content was lowered, indicating its essential role in mRNA encapsulation and endosomal escape.

Using cryo-TEM, we next examined how the freeze/thaw cycle and lipid composition influenced LNP morphology in F1-F6. In mRNA-LNPs, ionizable lipids like DLin-MC3-DMA typically form an electron-dense core by complexing with mRNA, cholesterol, and water, whereas peripheral blebs can harbor water and, in some cases, unencapsulated mRNA [31]. Consistent with other reports [32,33], both F1 and F4 showed a core-shell architecture with spherical shapes and smaller bleb-like regions. Notably, formulations with lower MC3 (i.e., higher DSPC) exhibited more pronounced blebs and diminished in vitro transfection. Although all LNPs underwent reduced particle concentration post-freeze/thaw, F1 (3% PS-80) still maintained a smaller particle size, robust freeze/thaw stability, and high luciferase expression and thus, was considered as the lead composition.

A key part of our optimization involved switching from a phosphate-sucrose (PSS) buffer to a tris-sucrose-salt (TSS) buffer. Phosphate-based systems frequently experience pH fluctuations (up to 3.5 pH units) upon freezing, which can degrade mRNA and alter LNP structure [10,34]. By contrast, TSS formulations were more “pH-resistant” and retained both structural and functional stability after long-term storage at −80 °C. Indeed, SC2-PS80 LNPs in TSS remained small in particle size (~192 nm), with high mRNA purity (61%) and robust in vitro transfection efficiency even after six months under frozen conditions, unlike their PSS counterparts, which aggregated and lost activity. However, SC2-PS80 LNPs stored in the liquid state (both 5 °C and 25 °C) showed a significant drop in their in vitro transfection following six months and might need further optimization to enhance their stability for long-term storage.

The in vivo immunogenicity experiments verified that SC2-PS80 LNPs stored at −80 °C in TSS elicited comparable anti-spike IgG binding antibodies and anti-SARS-CoV-2 neutralizing antibodies activity to freshly prepared formulations. By contrast, LNPs stored at 5 °C yielded negligible serologic responses, reflecting their poor in vitro transfection. These results demonstrate that formulating in TSS buffer with PS-80 as the PEG-lipid can not only redirect LNPs away from the liver but also preserve vaccine potency under frozen conditions. Finally, although we did not detect any visible injection-site reactions (e.g., swelling or limping) in our mouse studies, the detailed histopathological assessments or cytokine profiling remained to be comprehensively evaluated. Given PS-80’s extensive clinical use in approved biologics, we anticipate a favorable safety profile for the PS-80-LNP platform. Overall, to the best of our knowledge, this is the very first study highlighting the potential of this novel lipid composition containing PS-80 as the PEG-lipid for vaccine applications of mRNA-LNPs, with acceptable stability over six months when stored in a frozen state.

## 5. Conclusions

Using a stepwise optimization strategy, we developed and characterized a new PS-80-based mRNA-LNP vaccine formulation aimed at immune-induction against SARS-CoV-2. Our findings demonstrate that incorporating PS-80 as a branched PEG-lipid and formulating in a tris-sucrose-salt (TSS) buffer mitigates two major limitations often faced by mRNA-LNP vaccines: preferential liver uptake and suboptimal freeze/thaw and storage stability. Specifically, PS-80-based LNPs exhibited enhanced spleen tropism over liver accumulation, maintained high mRNA encapsulation and activity after over six months of storage at −80 °C, and elicited potent in vivo humoral immune responses.

Notably, we confirmed that 10% sucrose and ≥45 mM NaCl in a tris buffer was crucial to preserving particle size and mRNA integrity of PS-80-based LNPs during the freeze/thaw cycle, and that 3 mol% PS-80 balanced robust transfection efficiency with smaller, well-defined LNPs. Extending these findings to SARS-CoV-2 spike mRNA-LNPs (SC2-PS80 LNPs), we observed stable physicochemical properties and strong immunogenicity which were comparable to freshly prepared formulations after six months of frozen storage. Taken together, these results indicate that a PS-80-based mRNA-LNP platform can improve extrahepatic delivery, offer durable frozen stability in tris-sucrose-salt based platform, and drive robust immune induction. Such improvements have the potential to streamline both the manufacturing and storage of next-generation mRNA vaccines, ultimately contributing to more effective and accessible prophylactic strategies against viral pathogens, including SARS-CoV-2.

## Figures and Tables

**Table 1:** Lipid composition of the screened Fluc-PS80 LNP formulations.

Formulations	Lipid Components (Molar Percent Ratio)			
	Dlin-MC3-DMA	DSPC	Cholesterol	PS-80
F1	52	8	37	3
F2	50	10	37	3
F3	48	12	37	3
F4	52	8	38.5	1.5
F5	50	10	38.5	1.5
F6	48	12	38.5	1.5

**Table 2:** Panel of quality attributes and respective analytical techniques/assays used to evaluate the mRNA-LNP drug product quality.

Quality Attribute	Analytical Techniques/Assays
Visual appearance	Manual
pH	pH meter
Particle size	Dynamic light scattering (DLS) Electrophoretic mobility
Surface charge	
Particle concentration	Nanoparticle tracking analysis (NTA)
Encapsulation efficiency	RiboGreen^TM^ Assay
Particle morphology	Cryo-TEM
mRNA purity	Microchip capillary electrophoresis (MCE)
In vitro transfection efficiency Fluc-PS80 LNP SC2-PS80 LNP	Cell-based luminescence assay Immune staining and Flow cytometry
In vivo immunogenicity in mice	Luminex Spike Assay, Pseudovirus neutralization assay

**Table 3:** LNP formulations used for preliminary evaluation of the Fluc-PS80 LNPs versus DMG-PEG2K LNPs organ distribution.

LNP Formulation	Lipid Composition (MC3/DSPC/Chol/PEG-lipid mol%)	mRNA Buffer	Injection Route
1.5% DMG-PEG	52/8/38.5/1.5	10 mM Sodium Citrate, pH 5	IV
3% DMG-PEG	52/8/37/3	10 mM Sodium Acetate, pH 4.5	IV
1.5% DMG-PEG	52/8/38.5/1.5	10 mM Sodium Acetate, pH 4.5	IM
3%, DMG-PEG	52/8/37/3	10 mM Sodium Acetate, pH 4.5	IM
1.5% PS-80	52/8/38.5/1.5	10 mM Sodium Acetate, pH 4.5	IM
3% PS-80	52/8/37/3	10 mM Sodium Acetate, pH 4.5	IM

## Data Availability

The original contributions presented in this study are included in the article/Supplementary Material. Further inquiries can be directed to the corresponding author(s).

## Supplementary Materials

The following supporting information can be downloaded at https://www.mdpi.com/article/10.3390/pharmaceutics17040441/s1, Figure S1. Long-term storage stability results of Fluc-DMG-PEG LNPs formulated in PSS buffer following six months when stored at different temperatures. Figure S2. Long-term storage stability results of Fluc-DMG-PEG LNPs formulated in TSS buffer following six months when stored at different temperatures. Figure S3. Physicochemical characterization of Fluc-PS80 LNP formulations prepared in tris-sucrose (TS) and tris-sucrose containing 45 mM NaCl (TSS) buffers. Figure S4. Visual inspection of various Fluc-PS80 LNP formulations prepared in TSS buffer. Figure S5. Dynamic light scattering graphs depicting particle size and size distribution of Fluc-PS80 LNP formulations containing various lipid combinations prepared in TSS buffer. Figure S6. Long-term storage stability results of SC2-PS80 LNPs formulated in (a-b) PSS and (c-d) TSS buffers following six months when stored at different temperatures. Figure S7. Imaged capillary electrophoresis (iCE) of Fluc-PS80 LNPs and DMG-PEG LNPs.

## Abbreviations

The following abbreviations are used in this manuscript:

Cryo-TEM	Cryogenic transmission electron microscopy
DLin-MC3-DMA	[(6Z,9Z,28Z,31Z)-heptatriaconta-6,9,28,31-tetraen-19-yl] 4-(dimethylamino)butanoate
DMEM	Dulbecco’s Modified Eagle’s Medium
DMG-PEG2K LNP	Fluc mRNA-LNP containing DMG-PEG2K as the PEG-lipid
DMG-PEG2K	1,2-dimyristoyl-rac-glycero-3-methoxypolyethylene glycol-2000
DSPC	Distearoylphosphatidylcholine
FBS	Fetal bovine serum
FLuc mRNA	Firefly luciferase mRNA
Fluc-PS80 LNP	Firefly luciferase reporter mRNA-LNP
HEK-293 cells	Human embryonic kidney 293 cells
LNPs	Lipid nanoparticles
MCE	Microchip capillary electrophoresis
PS-80	Polysorbate-80
PSS	Phosphate-saline-sucrose buffer
pVNT50	50% pseudovirus neutralization titer
SARS-CoV-2	Severe acute respiratory syndrome coronavirus 2
SC2-PS80 LNP	Polysorbate 80-based SARS-CoV-2 mRNA-LNP
TSS	Tris-saline-sucrose buffer
