# Supplementary material for: A Polysorbate-Based Lipid Nanoparticle Vaccine Formulation Induces In Vivo Immune Response Against SARS-CoV-2
Source: Pharmaceutics. 2025 Mar 29;17(4):441. doi: 10.3390/pharmaceutics17040441 (PMC12030230; doi:10.3390/pharmaceutics17040441)
Supplement: Supplementary file 1 [file pharmaceutics-17-00441-s001.zip › pharmaceutics-3525384-supplementary.pdf]

pharmaceutics

Supplementary Material

A Polysorbate-Based Lipid Nanoparticle Vaccine Formulation Induces In Vivo Immune Response Against SARS-CoV-2

Aishwarya Saraswat [1,†], Alireza Nomani [1,*,†], Lin-Kin Yong [2], Jimmy Chun-Tien Kuo [3], Heather Brown [3], Muralikrishna Narayanareddygari [2], Avery Peace [4], Rizan Fazily [1], Timothy Blake [3], Christopher D. Petro [4], Bindhu Rayaprolu [1], Johanna Hansen [2], Amardeep Singh Bhalla [1] and Mohammed Shameem [1]

[1] Formulation Development Group, Regeneron Pharmaceuticals, Tarrytown, NY, USA
[2] Vaccine Technology, Regeneron Pharmaceuticals, Tarrytown, NY, USA
[3] Regeneron Genetic Medicines, Regeneron Pharmaceuticals, Tarrytown, NY, USA
[4] Infectious Diseases, Regeneron Pharmaceuticals, Tarrytown, NY, USA
* Correspondence: alireza.nomani@regeneron.com
† These authors contributed equally to this work.

1. Materials and Methods

1.1. Formulation buffer screening for long-term storage stability of Fluc LNPs containing DMG-PEG2K (Fluc-DMG-PEG LNPs)

Fluc-DMG-PEG LNPs containing the standard lipid composition of MC3/Cholesterol/DSPC/PEG-DMG (at molarity% of 50/10/37.5/2.5) were manufactured via microfluidic mixing using NanoAssemblr® Ignite™ system (Precision NanoSystems Inc.). For that, the lipid components were dissolved in 200 Proof absolute ethanol at a total lipid concentration of 50 mM, while Fluc mRNA (200 ug/mL) was diluted in 25 mM sodium citrate buffer pH 5.0. Lipids and Fluc mRNA solutions were both mixed at the previously optimized flow rate ratio of 3:1 (aqueous:organic), total flow rate of 12 mL/min, and N/P ratio 14. Following the microfluidic mixing, LNPs were diluted with either of the two buffers; phosphate-sucrose (PSS, containing 1X PBS, 10% sucrose, pH 7.4) or tris-sucrose-saline (TSS, containing 50 mM Tris, 45 mM NaCl, 10% sucrose, pH 7.4). Diluted LNPs were subjected to buffer exchange using ultrafiltration, then sterile-filtered by 0.2 um PVDF syringe filter and filled into 2R vials (Schott Pharma). LNPs were stored at different temperatures and samples were taken at various timepoints over six months for detailed characterizations. The analytical characterizations were performed for the samples as detailed in section 2.5.

2. Results

2.1. Formulation buffer screening for long-term storage stability of Fluc-DMG-PEG LNPs

Fluc-DMG-PEG LNPs were tested to compare two buffer types, the traditional PSS and the developed TSS buffers, for long-term stability at different storage conditions (-80 °C, 5 °C, 25 °C). Figure S1 represents the analytical characterization and *in vitro* potency results obtained for Fluc-DMG-PEG LNPs formulated in PSS buffer, when subjected to storage stability for a period of six months. As observed in Figure S1a-c, LNPs stored at -80 ºC showed a significant increase in their particle size from 59.4 ± 0.3 nm to 115.6 ± 0.84 nm, while the formulations stored at 5 ºC and 25 °C still showed no meaningful differences in size_following six months storage. Similarly, the polydispersity index of LNPs stored at -80 ºC increased from 0.042 ± 0.015 to 0.18 ± 0.014 as compared to the other formulations with no significant changes. This indicates that following the storage at frozen state and upon freeze-thaw, the Fluc-DMG-PEG LNPs in PSS did undergo fusion

and aggregations to show enlarged nanoparticles with less homogeneity when stored longer than a week at frozen state even in the presence of 10% sucrose as the cryoprotectant.

The zeta potential of all the tested formulations indicated neutral surface charge as the values ranged within ±10 mV with no significant changes over time. The encapsulation efficiency was found to be ≥85% for all the formulations at the tested time points. While the Fluc mRNA purity values for LNPs dropped at 5 ºC and 25 ºC conditions. It was observed that the values for 5 ºC samples dropped to 56.96 ± 1.47% and that for 25 ºC storage LNPs reduced to as low as 29.94 ± 1.21% over six months in PSS buffer (Figure S1d-e). Consequently, we observed a significantly reduced *in vitro* transfection efficiency for the formulations stored at all conditions (at_25 ºC, with the normalized mean luminescence intensity (MLI) dropping from 1.95 to 0.001 folds following three months, illustrating their drastic decreased potency). The normalized MLI values were also changed at a slower rate for the LNPs stored at -80 ºC (from 1.95 to 1.1 folds ratio to Lipofectamine), while that for 5 ºC storage LNPs reduced to 0.13 folds after six months (Figure S1f). Overall, these results indicated that the stability of Fluc-DMG-PEG LNPs prepared in PSS buffer was impacted in terms of their *in vitro* characteristics and/or potency when stored at all storage conditions (-80, 5, and 25 ºC) for six months, with the formulations stored at 25 ºC completely losing their activity within one month of storage.

Figure S2 illustrates the long-term storage stability results obtained for Fluc-DMG-PEG LNPs when formulated in the TSS buffer. As clearly seen, LNPs stored at -80 ºC showed improved stability in terms of their particle size (90.11 ± 0.87 nm) and polydispersity index (0.16 ± 0.01) when compared to the LNPs formulated in the PSS buffer. Formulations stored at 5 ºC and 25 ºC, nevertheless, exhibited a particle size of 60-70 nm and polydispersity index of ≤0.1 following six months (Figure S2a-b). The zeta potential and encapsulation efficiency of all Fluc-DMG-PEG LNP formulations in TSS resulted in no significant change over time when tested at different storage conditions (Figure S2c, e). The mRNA purity values for LNPs stored at 5 ºC dropped to 60.3 ± 0.4% over six months and that for 25 ºC storage LNPs reduced to as low as 34.7 ± 0.49% after three months (Figure S2d). These values are better than those obtained with LNPs prepared in PSS buffer.

Additionally, the *in vitro* transfection efficiency obtained for TSS-based Fluc-DMG-PEG LNPs was significantly higher than that observed for PSS-based formulations. Specifically, the normalized

MLI values for the LNPs stored at -80 ºC and 5 ºC were higher and remained unchanged over six months at 1.23 and 1.24 folds MLI compared to Lipofectamine, respectively. While the normalized MLI obtained for 25 ºC storage LNPs reduced to 0.008-fold after three months, which was significantly higher than that observed for LNPs formulated in the PSS buffer (Figure S2f). These results indicate that Fluc-DMG-PEG LNP has substantially higher stability in terms of their physiochemical properties and *in vitro* activity when prepared in TSS buffer, and -80 ºC (frozen state) being the most stable storage condition followed by 5 ºC (liquid form). LNP formulations stored at 25 ºC were slightly more stable when prepared in TSS buffer, however, this needs further optimization to enhance their drug product quality for long-term storage.

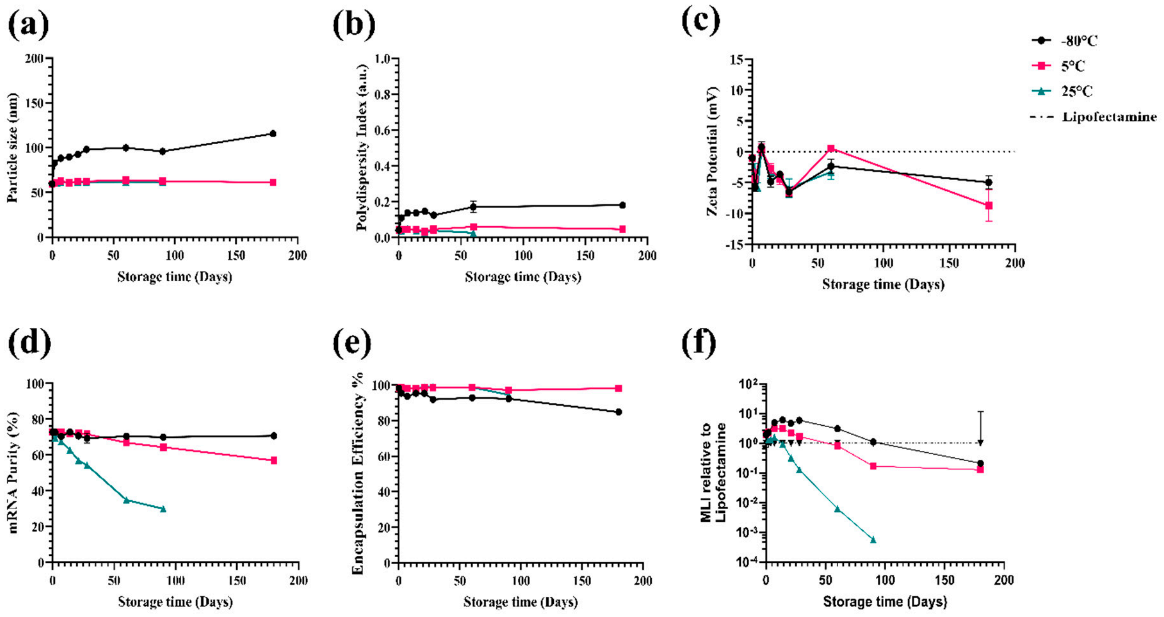

Figure S1. Long-term storage stability results of Fluc-DMG-PEG LNPs formulated in PSS buffer following six months when stored at different temperatures. Fluc-DMG-PEG LNPs stored at -80 ºC depicted increased particle size and polydispersity index, while those stored at 25 ºC demonstrated reduced mRNA purity and in vitro transfection efficiency overtime at all conditions with more prominent impact seen at 25 ºC followed by 5 ºC and -80 ºC. MLI: Mean Luminescence Intensity.

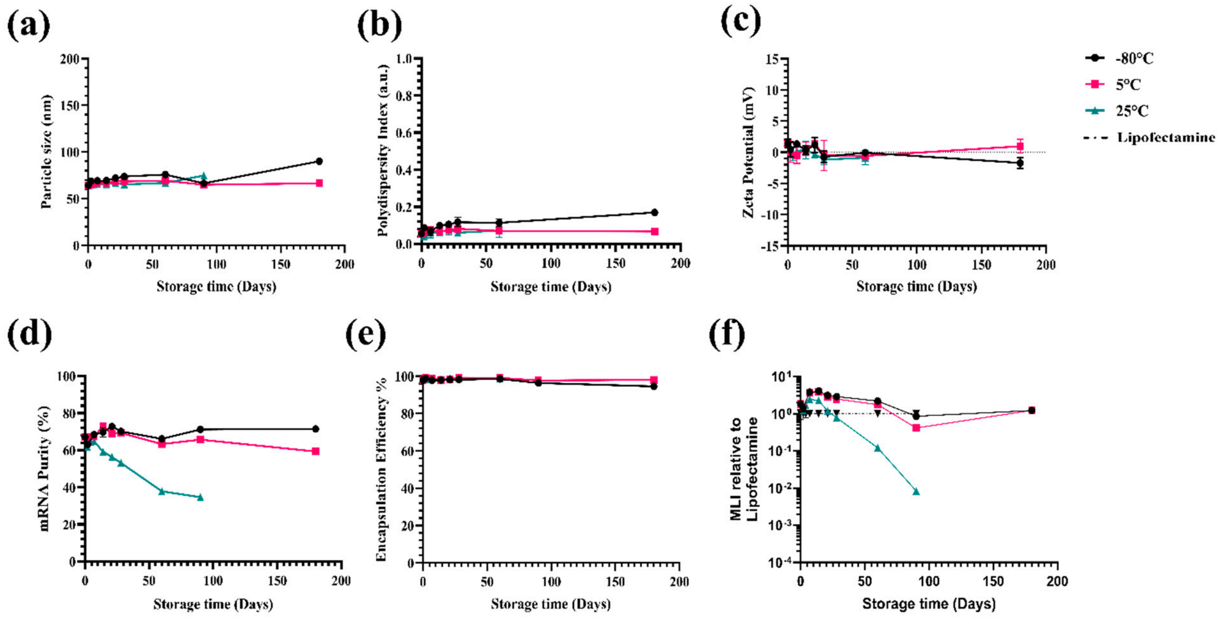

Figure S2. Long-term storage stability results of Fluc-DMG-PEG LNPs formulated in TSS buffer following six months when stored at different temperatures. Fluc-DMG-PEG LNPs stored at -80 ºC showed higher stability in terms of most physicochemical properties and in vitro transfection efficiency, while those stored at 25 ºC depicted reduced mRNA purity and in vitro transfection efficiency over time. MLI: Mean Luminescence Intensity.

2.2. Effect of salt on the physicochemical properties and stability of Fluc-PS80 LNPs

For this, we formulated Fluc-PS80 LNPs with 1.5 and 3 mol% PS-80 in TS (50 mM tris with 10% sucrose and without salt) and TSS buffers (TS with 45 mM NaCl) and assessed the quality attributes for fresh formulations as well as for those subjected to one overnight freeze-thaw cycle at -80 ºC. As seen in Figure S3, a substantial increase in particle size was observed for both 1.5 and 3 mol% PS80-containing formulations in the absence of salt after one freeze-thaw cycle. Subsequently, these formulations also showed a decrease in their *in vitro* transfection efficiency when compared to fresh formulations following subject to freeze-thaw. Therefore, the presence of salt was necessary to maintain a stable and potent PS-80 LNP formulation and further formulation development was carried out in the presence of salt for tris-based PS-80 LNP formulations.

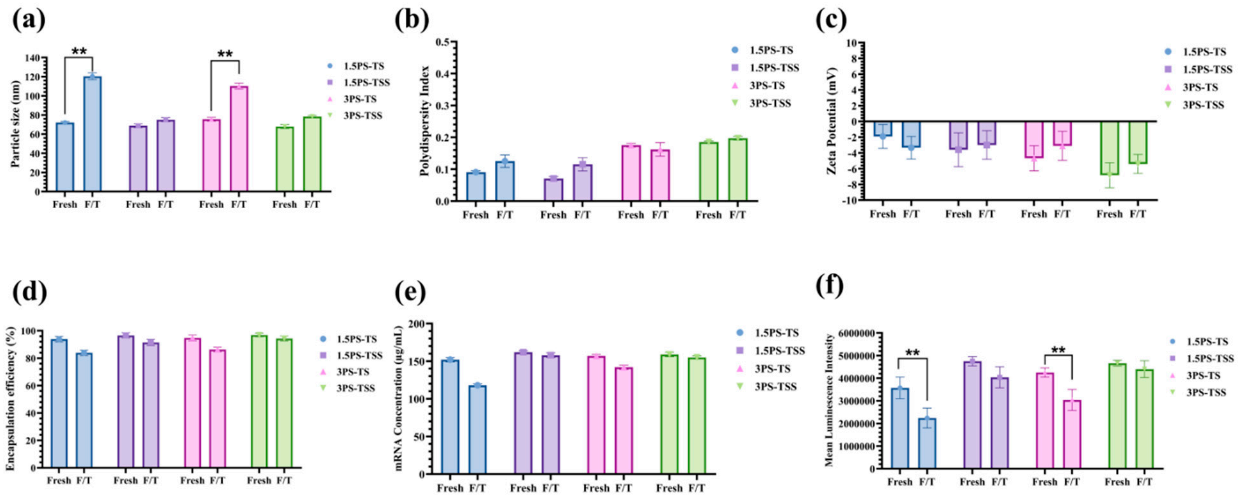

Figure S3. Physicochemical characterization of Fluc-PS80 LNP formulations prepared in tris-sucrose (TS) and tris-sucrose containing 45 mM NaCl (TSS) buffers, including **(a)** particle size, **(b)** polydispersity index, **(c)** zeta Potential, **(d)** encapsulation efficiency, **(e)** mRNA concentration, and **(d)** in vitro transfection efficiency analysis. A significant effect on the particle size and transfection efficiency was observed for the formulations lacking salt in their compositions (TS) when subjected to one freeze-thaw cycle. F/T: one freeze/thaw cycle. ** indicates a p-value of <0.001 (need to mention the stat test here). 1.5 PS: 1.5 mol% PS-80; 3PS: 3 mol% PS-80, TS: tris with 10% w/v sucrose, TSS: tris with 10% w/v sucrose and 45 mM sodium chloride.

2.3. Lipid combinations screening for Fluc-PS80 LNPs: Visual inspection

The visual appearance of LNPs is shown in Figure S4. Formulations F1-F3 containing 3 mol% PS-80 were more transparent as compared to F4-F6 formulations containing 1.5 mol% PS-80 which were slightly more turbid. None of the formulations showed any evidence of visual particles or aggregates at 5 ºC (Figure S4a-b). Following one freeze-thaw cycle at -80ºC, F1-F3 formulations did not show any change in their visual appearance, while F4-F6 formulations clearly exhibited increased turbidity, given these formulations contain a lower concentration of PS-80 (Figure S4c-d).

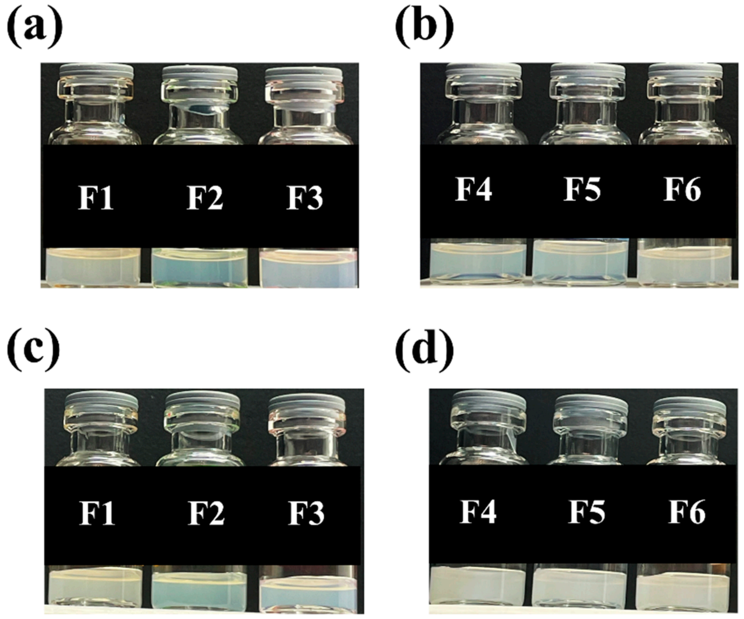

Figure S4. Visual inspection of various Fluc-PS80 LNP formulations prepared in TSS buffer. (a) F1-F3, **(b)** F4-6 fresh formulations. **(c)** F1-F3, **(d)** F4-6 formulations following one freeze-thaw cycle overnight at -80°C. F1-F3 formulations containing 3 mol% PS-80 demonstrated minimal change in their visual appearance, while F4-F6 formulations containing 1.5 mol% PS-80 showed increased turbidity.

2.4. Lipid combinations screening for Fluc-PS80 LNPs: Particle size distribution

Although all the formulations showed unimodal distribution, LNP formulations containing lower content of PS-80 (1.5 mol%) were more homogeneously dispersed with a narrow peak obtained for size distribution in comparison to the LNP formulations containing 3 mol% PS-80, as clearly seen in Figure S5a. Also, there were no substantial changes observed in the particle size and size distribution of any LNP formulations following one cycle of freeze-thaw (Figure S5b).

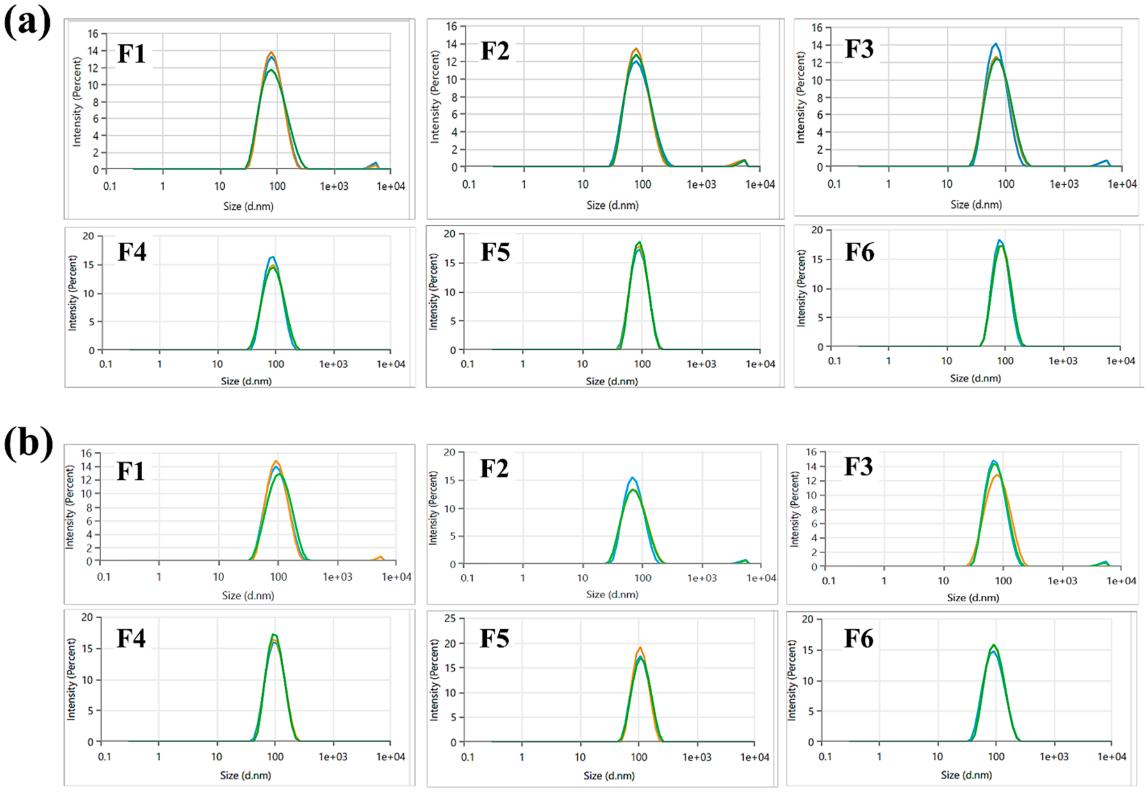

Figure S5. Dynamic light scattering graphs depicting particle size and size distribution of Fluc-PS80 LNP formulations containing various lipid combinations prepared in TSS buffer. (a) F1-F6 formulations showing particle size of ≤ 100 nm and monodispersed LNPs, **(b)** F1-F6 formulations demonstrating no significant change in their particle size or size distribution following freeze-thaw.

2.5. Formulation buffer screening for long-term storage of SC2-PS80 LNPs

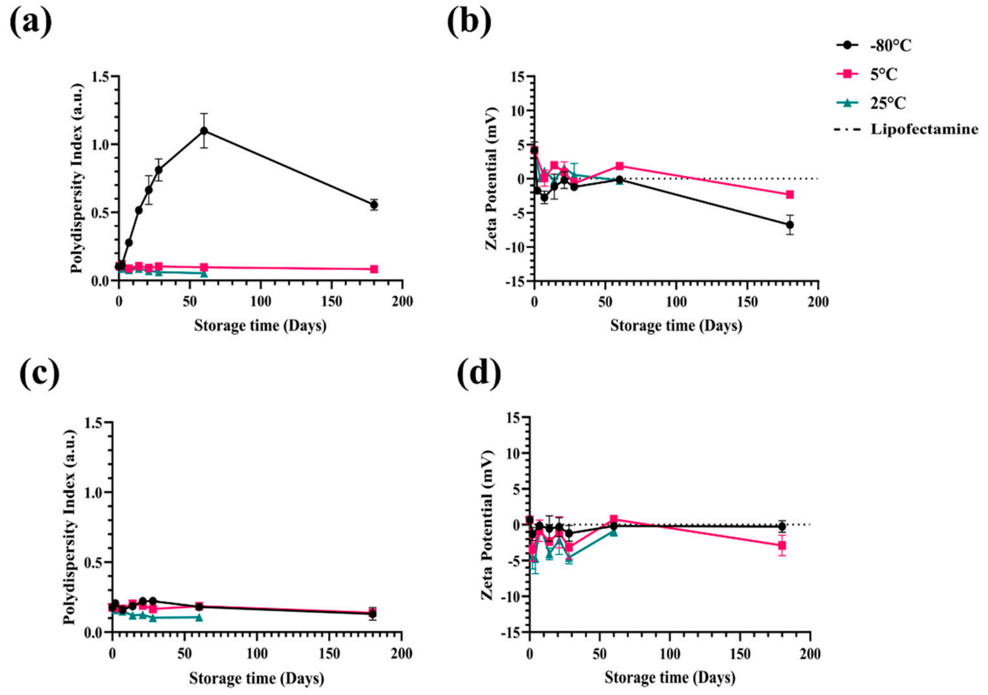

Figure S6. Long-term storage stability results of SC2-PS80 LNPs formulated in (a-b) PSS and (c-d) TSS buffers following six months when stored at different temperatures. A significant increase in polydispersity index was seen for PSS-containing formulation stored at –80 ºC in comparison to the TSS-containing formulation over six months.

2.6. Imaged capillary electrophoresis (iCE) of Fluc-PS80 LNPs and Fluc-DMG-PEG LNPs

Imaged capillary electrophoresis (iCE) was performed using an iCE3™ instrument (ProteinSimple). Briefly, 1 µg of Fluc mRNA from PS80-LNP or DMG-PEG LNP (at a similar N:P ratio of 14) was mixed with pre-optimized ampholytes (pH 3-10 and pH 5-8), 0.5% methylcellulose, and 10% glycerol in a final volume of 160 µL. Isoelectric point (pI) markers (5.85 and 8.40) were included to normalize the pH gradient. Approximately 120 µL of the prepared sample was loaded into the icIEF cartridge, where focusing proceeded for 1 min at 1500 V/cm (pre-focus) followed by 8 min at 3000 V/cm. UV absorbance was monitored at 280 nm to detect the focused LNP peaks, and resulting electropherograms were aligned to the pI markers for the final analysis of each sample's isoelectric point.

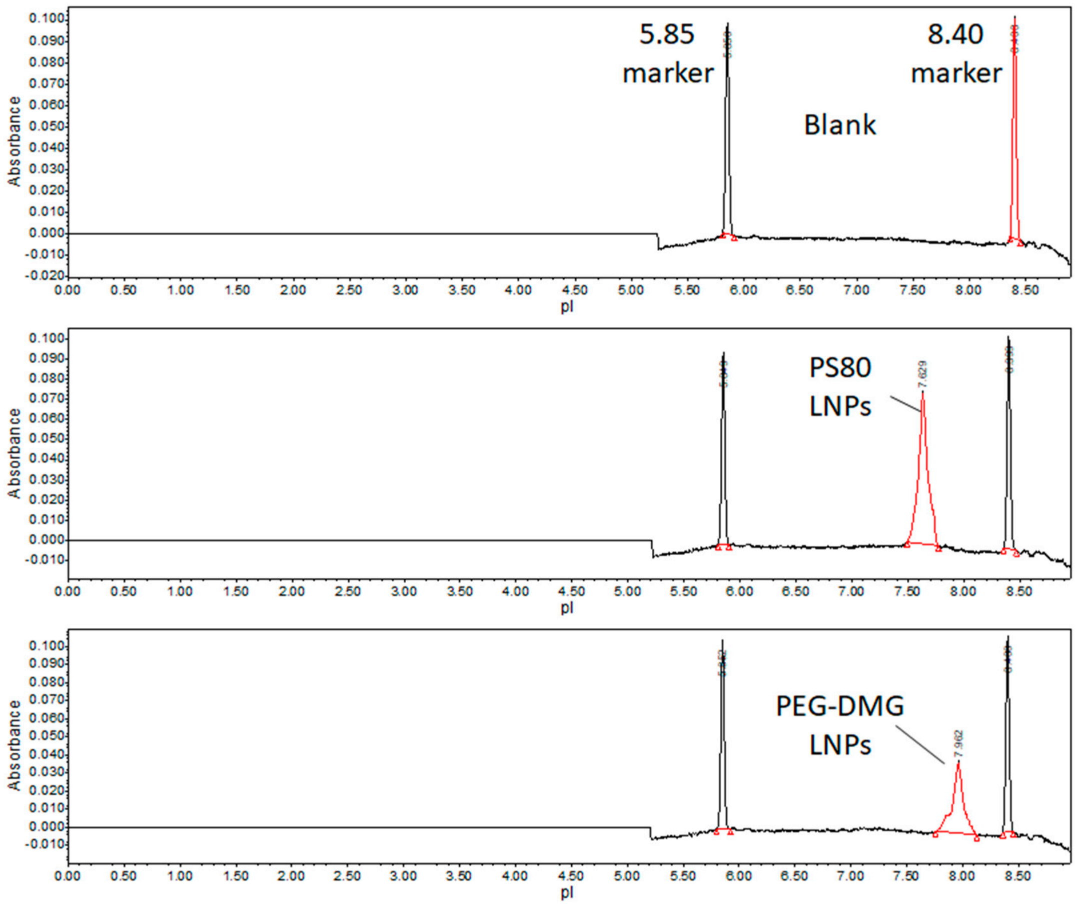

Figure S7. Imaged capillary electrophoresis (iCE) of Fluc-PS80 LNPs and DMG-PEG LNPs.
